# Spinal Fluid Lactate Dehydrogenase Level Differentiates between Structural and Metabolic Etiologies of Altered Mental Status in Children 

**Published:** 2015

**Authors:** Nahid KHOSROSHAHI, Parastoo ALIZADEH, Mehdi KHOSRAVI, Peyman SALAMATI, Kamyar KAMRANI

**Affiliations:** 1 Department of Pediatric Neurology, Tehran University of Medical Sciences, Tehran, Iran; 2 Department of Medicine, University of Kentucky, Lexington, Kentucky, USA; 3 Department of Community Medicine, Tehran University of Medical Sciences, Tehran, Iran; 4 Department of Neonatology, Tehran University of Medical Sciences, Tehran, Iran

**Keywords:** Lactate dehydrogenase, Cerebrospinal fluid, Altered mental status, Brain imaging

## Abstract

**Objective:**

Altered mental status is a common cause of intensive care unit admission in children. Differentiating structural causes of altered mental status from metabolic etiologies is of utmost importance in diagnostic approach and management of the patients. Among many biomarkers proposed to help stratifying patients with altered mental status, spinal fluid lactate dehydrogenase appears to be the most promising biomarker to predict cellular necrosis.

**Materials & Methods:**

In this cross sectional study we measured spinal fluid level of lactate dehydrogenase in children 2 months to 12 years of age admitted to a single center intensive care unit over one year. Spinal fluid level of lactate dehydrogenase in 40 pediatric cases of febrile seizure was also determined as the control group.

**Results:**

The study group included 35 boys (58.3%) and 25 girls (41.7%). Their mean age was 2.7+/-3 years and their mean spinal fluid lactate dehydrogenase level was 613.8+/-190.4 units/liter. The control group included 24 boys (55.8%) and 19 girls (44.2%). Their mean age was 1.3+/-1.2 years and their mean spinal fluid lactate dehydrogenase level was 18.9+/-7.5 units/liter. The mean spinal fluid lactate dehydrogenase level in children with abnormal head CT scan was 246.3+/-351.5 units/liter compared to 164.5+/-705.7 in those with normal CT scan of the head (p=0.001).

**Conclusion:**

Spinal fluid lactate dehydrogenase level is useful in differentiating structural and metabolic causes of altered mental status in children.

## Introduction

Altered mental status is a common cause of intensive care unit admission in children (1). This condition may be caused by structural brain abnormalities (ischemia, tumors, hemorrhage, abscess or meningitis) or nonstructural brain abnormalities (hypoglycemia, hyponatremia, dehydration, hepatic encephalopathy or uremic encephalopathy) (2). The routine diagnostic measures may not accurately distinguish between structural and metabolic etiologies of altered mental status (3) and imaging modalities are generally required for completion of diagnostic work up (4). In many parts of the world, advanced imaging is not readily available and emergency department physician needs to rely on his or her clinical acumen to manage such patients (5). 

Recent studies have focused on serum and cerebrospinal fluid (CSF) markers such as creatine kinase, creatine kinase-BB, alanine trans aminase, aspartate trans aminase, or lactate dehydrogenase (LDH) to help distinguish structural and metabolic causes of altered mental status. In hypoxic and ischemic brain injuries LDH seems to be the most reliable among aforementioned biomarkers (6). Increase in spinal fluid LDH level is an indication of either neuronal or glial cell injury (5). Wroblewski et al first described the clinical importance of elevated spinal fluid LDH in 1957. In 2008, Vazquez et al showed spinal fluid LDH level bellow 40 units/liter in adult critically ill patients with non traumatic altered mental status is indicative of a metabolic derangement (5). 

Prior studies have indicated that spinal fluid LDH level is higher in cases of bacterial and tuberculous meningitis when compared to LDH levels in patients with aseptic meningitis (7,8,9,10,11,12,13,14,15,16,17) . 

Considering the lack of data in pediatric population, in the current study, we documented the spinal fluid LDH level of children from 2 months of age to 12 years old with altered mental status and its association with the etiology of change in level of consciousness. 

## Materials & Methods

We undertook this study to determine CSF lactate dehydrogenase level in patient with decreased level of consciousness resulting from either structural or metabolic insults to central nervous system (CNS). We defined decreased level of consciousness as lack of awareness of surrounding environment in a spectrum from somnolence to deep coma. We hypothesized that CSF LDH level will defer in structural and metabolic CNS insults. 

All patients from 2 month to 12 years of age who presented to Bahrami hospital (Tehran, Iran) intensive care unit during a 12 month period in 2009 with decreased level of consciousness were included in the study. Patient with recent trauma, known brain tumor, bleeding diathesis, or skin infection at the site of potential lumbar puncture were excluded from the study. Also patients with a contraindication to lumbar puncture such as those with increased intracranial pressure or signs of impending cerebral herniation were excluded. Additionally, patients with bloody CSF sample, and terminally ill subjects were also excluded. 

All subjects underwent a complete history and physical exam. All CSF specimens were sent immediately to lab for measuring LDH levels and other routine analysis. All subjects also had imaging of the brain either in form of CT scan or MRI. 

For the control group, 43 children with complex febrile seizure (e.g:focal,prolonged,developmental delay,…) admitted to Bahrami hospital (Tehran, Iran) were evaluated. They also underwent lumbar puncture and brain imaging. LDH levels in CSF samples were measured. 

An independent neurologist, blinded from CSF measurements, reviewed the brain imaging of all patients and stratified them based on presence of structural brain abnormalities. 

The data was analyzed by SPSS 17 software (IBM, 2008). Kolmogorov-Smirnov and Mann-Whitney U test were used for analysis. 

## Results

There were 35 boys (58.3%) and 25 (41.7%) girls in the study group. The mean age in study group subjects was 2.68+/-3.01 years. The youngest study group subject was 2 months old and the oldest was 12 years old. There were 24 boys (55.8%) and 19 girls (44.2%) in the control group. The mean age in control group was 1.16+/-1.25 years. The youngest control group subject was 2 months old and the oldest was 7 years old. 

The mean CSF LDH level in study group was 190.39+/- 613.84 IU/L (ranging from 8 to 4548 IU/L). The mean CSF LDH level in control group was 18.89+/-7.49 IU/L (ranging from 5 to 39 IU/L). Most prevalent diagnoses in study group were biochemical abnormalities 16.7% (10 patients) and metabolic disturbances 13.3% (8patients). In the study group, 41 subjects had normal brain imaging (68.3%)in metabolic group and 19 subjects (31.7%) had abnormal brain imaging in structural group. 

There were 12 boys (63.2%) and 7 girls (36.8%) in the group with abnormal brain imaging. The mean age in the group with abnormal imaging was 3.97+/-4.03 years. Mean CSF LDH level in the group with abnormal brain imaging was 246.26+/-351.45 IU/L (refer to table 1).

The most prevalent diagnosis in the group with abnormal brain imaging was intracranial hemorrhage (refer to table 1). 

There were 23 boys (56.1%) and 18 girls in the group with normal brain imaging. Mean CSF LDH level in the group with normal brain imaging was 164.49+/-705.71 IU/L (refer to table 1). The most prevalent diagnosis in the group with normal brain imaging was biochemical abnormalities (refer to table 1). 

The mean CSF LDH level in group with abnormal brain imaging was significantly larger than the group with normal brain imaging (p=0.001). 

There were 7 subjects in the study group with bacterial meningitis (mean age 2.09+/-2.51 years). The mean CSF LDH level in this group was 804.28+/-1655.20 IU/L. There were 5 subjects in study group with aseptic meningitis (mean age 2.68+/-2.23 years). The mean CSF LDH level in this group was 29.5+/-12.04 IU/L. There was only one patient with tuberculous meningitis whose CSF LDH level was 1122 IU/L (refer to table 3). 

## Discussion

The CSF biomarkers are focus of recent studies to assess etiology of decreased level of consciousness (5). In this study we were able to show that CSF LDH levels are significantly higher in patients with decreased level of consciousness and abnormal brain imaging. Prior studies have also found CSF LDH of importance. Wroblewski, et al, first described the clinical importance of elevated LDH level in CFS fluid (19). Parakh, et al, described elevation of CSF LDH in ischemic stroke patients (19). Lampel, et al, described the prognostic value of CSF LDH level in ischemic stroke patients (20). Hall, et al, also found elevation of CSF LDH levels in infants that died from anoxic encephalopathy (21). Nussinovitch, et al, were able to show CSF LDH levels increase in cases of brain injury, obstructive encephalopathy and other neurodevelopmental abnormalities (22). Engelke, et al, also showed that in newborns with intracranial hemorrhage, the CSF LDH level correlates with severity of intracranial hemorrhage as measured on CT scan. 

Most researches above , done in adults with limited sample size and also most executed in vascular lesions and meningitis . Our study has been accomplished uniquely in children and also with large sample size and variety of brain structural lesions and we had similar results concerning to raised LDH in CSF as above researches . 

**Table 1:** Lactate Dehydrogenase Level in Study Group Stratified By Imaging Results.

	**Minimum LDH Level (IU/L)**	**Maximum LDH Level (IU/L)**	**Mean LDH Level (IU/L)**	**Std**
**Normal brain imaging**	8	4548	164.49	705.71
**Abnormal brain imaging**	13	1122	246.26	351.45

**Table 2:** Final Diagnosis in Study Group Stratified by Imaging Results.

	**Diagnosis**	**No. of subjects**	**Percentage**
**Subjects with normal brain imaging**	Aseptic meningitis	5	12.2
	Bacterial meningitis	7	17.1
	Encephalitis	1	2.4
	Seizure disorders	6	14.6
	Sepsis	2	4.9
	Dehydration	1	2.4
	Drug toxicity	2	4.9
	Metabolic disturbances	6	14.6
	Biochemical abnormalities	10	24.4
	Pseudotumor Cerebri†	1	2.4
	Total	41	100
**Subjects with abnormal brain imaging**	Tuberculous Meningitis	1	5.3
	Metabolic disturbances* (inborn error of metabolism)	2	10.5
	Brain tumors	2	10.5
	Intracranial Hemorrhage	4	21.1
	Subdural hematoma	1	5.3
	Hypoxic/ischemic encephalopathy	2	10.5
	Stroke	1	5.3
	Brain abscess	1	5.3
	Central pontine myelinolysis	1	5.3
	ADEM/ANEC	3	15.8
	Multiple sclerosis	1	5.3
	total	19	100

* Including one patient with type I glutaric aciduria with subdural effusion and frontal atrophy on CT scan and a patient with Zellweger syndrome with communicative hydrocephalus PVL and organized intraventricular hemorrhage on CT scan.

† A patient with ultimate diagnosis of pseudotumor Cerebri, who had altered level of consciousness because of antiemetic drug usage.

**Table 3:** CSF LDH Levels Stratified by Final Diagnosis

**Diagnosis**	**No. of subjects**	**Minimum LDH Level (IU/L)**	**Maximum LDH Level (IU/L)**	**Mean LDH Level (IU/L)**	**Std**
**Aseptic Meningitis**	5	29	45	29.5	12.04
**Bacterial meningitis**	7	18	4548	804.28	1655.2
**Tuberculous meningitis**	1	1122	1122	1122	0
**Encephalitis**	1	15	15	15	0
**Epilepsy**	6	8	34	17.51	8.86
**Sepsis**	2	10	15	12.5	3.53
**Dehydration**	1	12.6	12.6	12.6	0
**Drug toxicity**	2	10	24	17	9.89
**Metabolic disturbances** **(inborn error of metabolism)**	8	16	132	56	43.12
**Biochemical abnormalities**	10	13	108	40.62	27.67
**Pseudotumor Cerebri†**	1	17	17	17	0
**Brain tumors**	2	231	964	597.5	518.3
**Intracranial Hemorrhage**	4	13	164	73.5	64.84
**Subdural hematoma**	1	28	28	28	0
**Hypoxic/anoxic encephalopathy**	1	96	96	96	0
**Stroke**	1	82	82	82	0
**Brain abscess**	1	73	73	73	0
**Central pontine myelinosis**	1	62	62	62	0
**Hydrocephaly**	1	619	619	619	0
**ADEM/ANEC**	3	38	819	306	444.41
**Multiple sclerosis**	1	94	94	94	0

Our study is limited by the fact that lumbar puncture and imaging studies were only done in patients that were able to tolerate these procedures clinically and unstable patients were excluded from the study. We were limited by ethical and safety constraints but we included all patients that were otherwise eligible in the study. The lack of uniformity in imaging modalities used in the study might result in bias. We had a blinded neurologist review all the imaging studies to have uniformity in interpretation of the results. 

Our study suggests CSF LDH level may help separate structural causes of decreased level of consciousness from metabolic etiologies in children. 

**Conflict of Interest: **The authors have no conflicts of interest with any companies or organizations whose products or services may be discussed in this article. 

The manuscript represents original work that is not being considered or has been accepted for publication elsewhere. 

## Custom

P.Alizadeh helped in data gathering, preparing a database, article searching, and writing the manuscript. N.Khosroshahi planed the study, confirmed the patients’ diagnosis, helped in article searching and supervised the project. P.Salmati helped in analyzing the article and M.Khosravi helped in professional translation of manuscript. K.Kamrani helped in article searching and writing manuscript. All authors read and approved the final manuscript.
